# Time-Dependent DNA Origami Denaturation by Guanidinium Chloride, Guanidinium Sulfate, and Guanidinium Thiocyanate

**DOI:** 10.3390/ijms23158547

**Published:** 2022-08-01

**Authors:** Marcel Hanke, Niklas Hansen, Emilia Tomm, Guido Grundmeier, Adrian Keller

**Affiliations:** Technical and Macromolecular Chemistry, Paderborn University, Warburger Str. 100, 33098 Paderborn, Germany; marcelha@mail.uni-paderborn.de (M.H.); niklashansen94@aol.de (N.H.); emiliat@mail.uni-paderborn.de (E.T.); g.grundmeier@tc.uni-paderborn.de (G.G.)

**Keywords:** DNA origami, DNA nanotechnology, guanidinium, denaturation, atomic force microscopy

## Abstract

Guanidinium (Gdm) undergoes interactions with both hydrophilic and hydrophobic groups and, thus, is a highly potent denaturant of biomolecular structure. However, our molecular understanding of the interaction of Gdm with proteins and DNA is still rather limited. Here, we investigated the denaturation of DNA origami nanostructures by three Gdm salts, i.e., guanidinium chloride (GdmCl), guanidinium sulfate (Gdm_2_SO_4_), and guanidinium thiocyanate (GdmSCN), at different temperatures and in dependence of incubation time. Using DNA origami nanostructures as sensors that translate small molecular transitions into nanostructural changes, the denaturing effects of the Gdm salts were directly visualized by atomic force microscopy. GdmSCN was the most potent DNA denaturant, which caused complete DNA origami denaturation at 50 °C already at a concentration of 2 M. Under such harsh conditions, denaturation occurred within the first 15 min of Gdm exposure, whereas much slower kinetics were observed for the more weakly denaturing salt Gdm_2_SO_4_ at 25 °C. Lastly, we observed a novel non-monotonous temperature dependence of DNA origami denaturation in Gdm_2_SO_4_ with the fraction of intact nanostructures having an intermediate minimum at about 40 °C. Our results, thus, provide further insights into the highly complex Gdm–DNA interaction and underscore the importance of the counteranion species.

## 1. Introduction

The fundamental physicochemical properties of DNA origami nanostructures and their interactions with various chemical and biological species and environments have attracted considerable attention over the past decade. Many of those investigations were motivated by the comparably low stability of DNA origami nanostructures in physiological media that have low concentrations of stabilizing Mg^2+^ ions and often contain DNA-digesting enzymes [1,2,3,4,5,6,7]. Other studies subjected DNA origami nanostructures to conditions relevant for lithographic processing [8,9,10] and long-term cryostorage [11,12,13], exposed them to ionizing radiation [14,15,16], and investigated their interactions with various ions [17,18,19], therapeutic drugs [20,21,22], and biomolecules [23,24,25]. Several of these works found that the behavior and interactions of DNA origami nanostructures under such conditions may differ distinctly from those of simple double-stranded (ds) DNA, which may lead to surprising and sometimes counterintuitive observations [16,17,19,21,23]. An important factor in this regard appears to be the dense packing of double helices inside a DNA origami, which may result in enhancement effects that couple comparatively small molecular transitions to macroscopic changes at the nanostructure level that can easily be detected, for instance, by atomic force microscopy (AFM) [14,15,26,27]. This renders DNA origami nanostructures powerful tools for gaining deeper insights into the chemical and physical mechanisms that control DNA structure, stability, and denaturation.

In our previous studies, we investigated the denaturation of DNA origami nanostructures by chaotropic guanidinium (Gdm) salts [28,29,30], which are widely used in biotechnology and biophysics as potent protein denaturants [31]. In general, the denaturation of a polypeptide chain is associated with a gain in conformational entropy, which is counteracted by an entropy loss of the surrounding water molecules resulting from the increase of solvent-excluded volume upon unfolding [32]. Both effects can be modulated by the addition of salts. The Gdm^+^ ion possesses flat hydrophobic faces that can participate in hydrophobic interactions. However, it can also participate in directional H-bonding via its three NH_2_ groups. Therefore, Gdm^+^ denatures protein secondary structure by competing for H-bonds and is additionally capable of stacking with nonpolar side chains, particularly those containing planar aromatic groups [33]. This denaturing effect of the Gdm^+^ cation may be modulated and even overcompensated by its counteranions, for instance, due to direct interactions with water molecules that result in the entropy loss upon denaturation becoming larger than the gain in conformational entropy of the unfolded polypeptide chain [32]. However, the molecular mechanisms involved in Gdm^+^–protein interactions are still a topic of intense research [34,35], and only few studies so far have attempted to elucidate its effects on dsDNA [36,37]. Our previous investigations revealed that Gdm^+^-induced DNA origami denaturation is governed by a complex interplay among the denaturing Gdm^+^ ions, the environmental conditions such as temperature and Gdm^+^ concentration [28], the counteranion species [30], and the presence of additional cations, which was found to induce more severe denaturation [29]. The counteranion species in particular was found to have a surprisingly strong influence on the denaturant activity of Gdm^+^. Using a combination of in situ circular dichroism (CD) spectroscopy and ex situ AFM, a thermodynamic model of DNA origami denaturation by guanidinium chloride (GdmCl) and guanidinium sulfate (Gdm_2_SO_4_) was derived on the basis of principle component analysis (PCA) and iterative target test factor analysis (ITTFA) [30]. The results showed that Gdm^+^-induced DNA origami denaturation proceeds via three successive state transitions involving an intermediate pre-melding state. Remarkably, this complex denaturation was further found to be driven by heat capacity changes, which are modulated by the counteranions via altered wetting properties of the hydrophobic DNA surface regions, particularly in the grooves. This was attributed to the presence of more water-like and less charged hydration shells in GdmCl and the more pronounced ion pairing in Gdm_2_SO_4_, in accordance with molecular dynamics (MD) simulations. Transfer of Gdm^+^ from GdmCl bulk solution to the DNA base stack upon heating, thus, results in a stronger increase in the number of ordered low-entropy water networks around the DNA origami nanostructures compared to Gdm_2_SO_4_.

In the present work, we extend our previous investigations and focus on the time dependence of DNA origami denaturation by GdmCl, Gdm_2_SO_4_, and guanidinium thiocyanate (GdmSCN). These three counterion pairings of the chaotropic Gdm^+^ cation cover the whole range of the anionic Hofmeister series [38]. Here, SO_4_^2−^ is located at the kosmotropic end, whereas Cl^−^ is found in the middle. SCN on the other hand is the most chaotropic anion in the Hofmeister series, which is reflected in the exceptionally strong denaturant activity of the chaotropic–chaotropic ion pair GdmSCN [34,39]. The effect of these three Gdm salts on the structural integrity of DNA origami triangles was assessed in 2 M solutions of the salts at different temperatures in 15 min intervals by ex situ AFM over a time course of 90 min. In general, we found that GdmSCN is the most potent DNA origami denaturant, which, at this comparably low concentration, could already cause complete DNA origami denaturation. Furthermore, while under moderately to strongly denaturing conditions, DNA origami denaturation occurred within the first 15 min of Gdm^+^ exposure, much slower DNA origami denaturation was observed under weakly denaturing conditions such as for Gdm_2_SO_4_ at 25 °C. Lastly, by choosing a wider temperature range, we were also able to observe a novel non-monotonous temperature dependence of DNA origami denaturation in Gdm_2_SO_4_ with the fraction of intact DNA origami nanostructures having an intermediate minimum at a temperature around 40 °C. Our results, thus, highlight the complexity of the Gdm^+^–DNA interaction and underscore the importance of the counteranion species, which may lead to complex and unexpected time and temperature dependencies of the Gdm^+^-induced DNA origami denaturation.

## 2. Results

### 2.1. Guanidinium Chloride (GdmCl)

**Figure 1:**
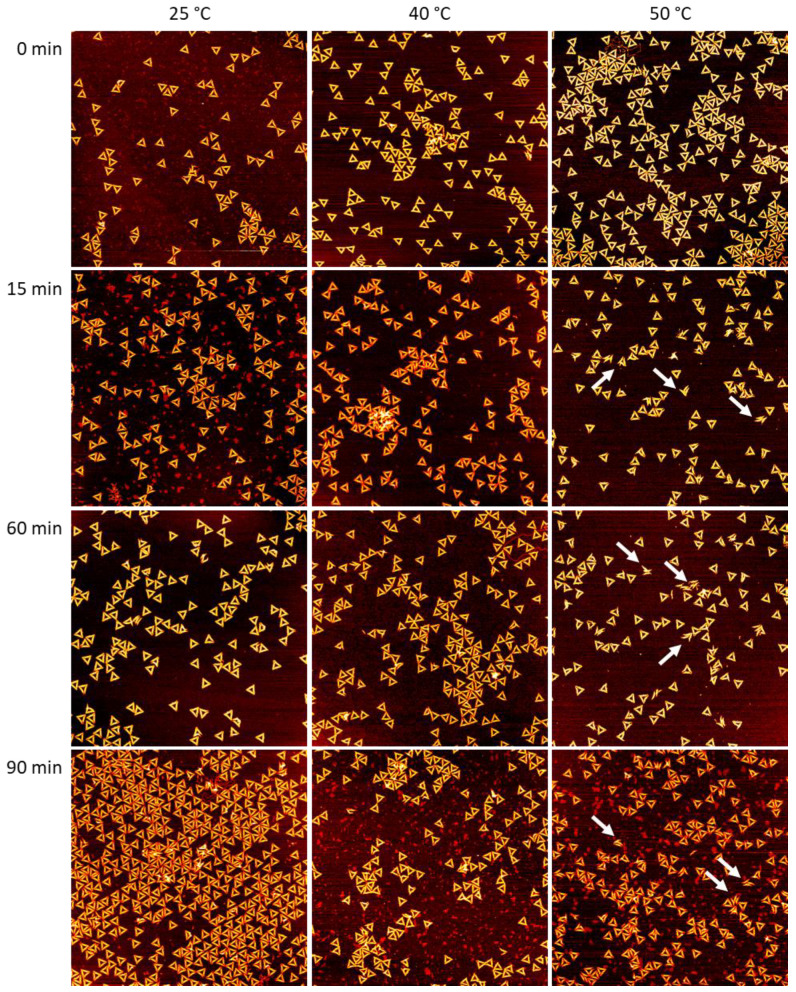
AFM images of DNA origami triangles after incubation in 2 M GdmCl at different times and temperatures. Images have a size and height scale of 3 × 3 µm^2^ and 2 nm, respectively. The white arrows indicate collapsed triangles that disintegrated by rupture at the vertices. For additional AFM images, see Figures S1–S3.

For investigating the time dependence of DNA origami denaturation in the different Gdm salts, we exposed DNA origami triangles [40] to comparably low Gdm salt concentrations of 2 M. The Rothemund triangle is one of the most studied DNA origami nanostructures, particularly with regard to its stability under various conditions [1,2,5,10,11,12,14,16,17,20,24,25,41,42,43,44,45], including the presence of molar concentrations of Gdm salts [28,29,30]. It is composed of three trapezoids of parallel double helices that are connected to each other via one scaffold crossover and four bridging staples. For exposure to 2 M GdmCl, only low to moderate fractions of damaged DNA origami triangles have been observed in the temperature range between 23 and 42 °C [30]. However, in the current work, we extended the temperature range slightly and recorded AFM images of the DNA origami triangles after incubation at 25, 40, and 50 °C, in order to assess a broader dynamic range in the observable damage. As can be seen in the AFM images in Figure 1, intact DNA origami triangles could be observed for all temperatures and incubation times. Note that 0 min incubation refers to the freshly prepared samples at room temperature before incubation at the desired temperature (see Section 4.2). Closer inspection also revealed some damaged triangles, which came apart at the vertices due to dissociation of the short bridging staples that connect the three trapezoids [28,30,41] (see white arrows in Figure 1). Note that this kind of damage is also observed in freshly assembled DNA origami triangles without any exposure to denaturing conditions [11]. However, damaged DNA origami nanostructures appeared rather rarely at 0 min, whereas they seemed to become more prominent at longer incubation times, particularly at 50 °C.

**Figure 2:**
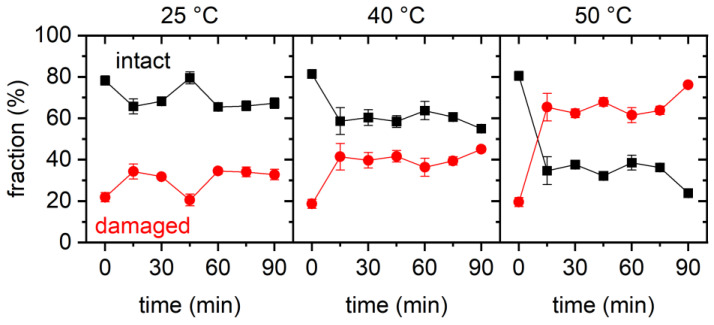
Results of the statistical analysis of the AFM images for 2 M GdmCl. Each data point represents the average of three AFM images with the standard deviations given as error bars.

These qualitative observations are further substantiated in the results of the statistical analyses of the AFM images shown in Figure 2. Here, the fractions of intact and damaged DNA origami are plotted as a function of incubation time for all three temperatures. The fractions were determined by manual counting with the classification “damaged” applying to all DNA origami nanostructures with compromised triangular shapes, ranging from triangles with a single ruptured vertex to completely disintegrated structures. For all three temperatures, we observed an initial decrease in the fraction of intact DNA origami within the first 15 min of incubation. At 25 °C, the fraction of intact DNA origami dropped only slightly from initially about 80% to about 65%, while larger drops to about 60% and less than 40% were observed for 40 and 50 °C, respectively. This general trend agrees fairly well with previously reported observations [30]. For incubation times exceeding 15 min, the fractions of intact DNA origami nanostructures remained more or less constant but showed some random fluctuations that can be attributed to sample-to-sample variations. At a temperature of 50 °C, an additional drop in the fraction of intact DNA origami was observed between 75 and 90 min incubation. However, since this drop had a similar magnitude to the maximum variation observed in the 25 °C data at intermediate times, we attribute this behavior to random fluctuations and not to the onset of a second, late-stage denaturation phase.

Summing up our observations for GdmCl, it seems that, under the chosen conditions, DNA origami damage occurred mostly within the first 15 min of GdmCl exposure. Longer incubation times up to 90 min resulted neither in more damaged DNA origami nor in more severe damage. The observed degree of DNA origami damage was comparably low and mostly consisted of ruptured vertices of the DNA origami triangles, while the trapezoids remained almost completely intact. Furthermore, the fraction of damaged DNA origami nanostructures increased with temperature. All this is in fair agreement with previous investigations [28,30].

### 2.2. Guanidinium Sulfate (Gdm_2_SO_4_)

**Figure 3:**
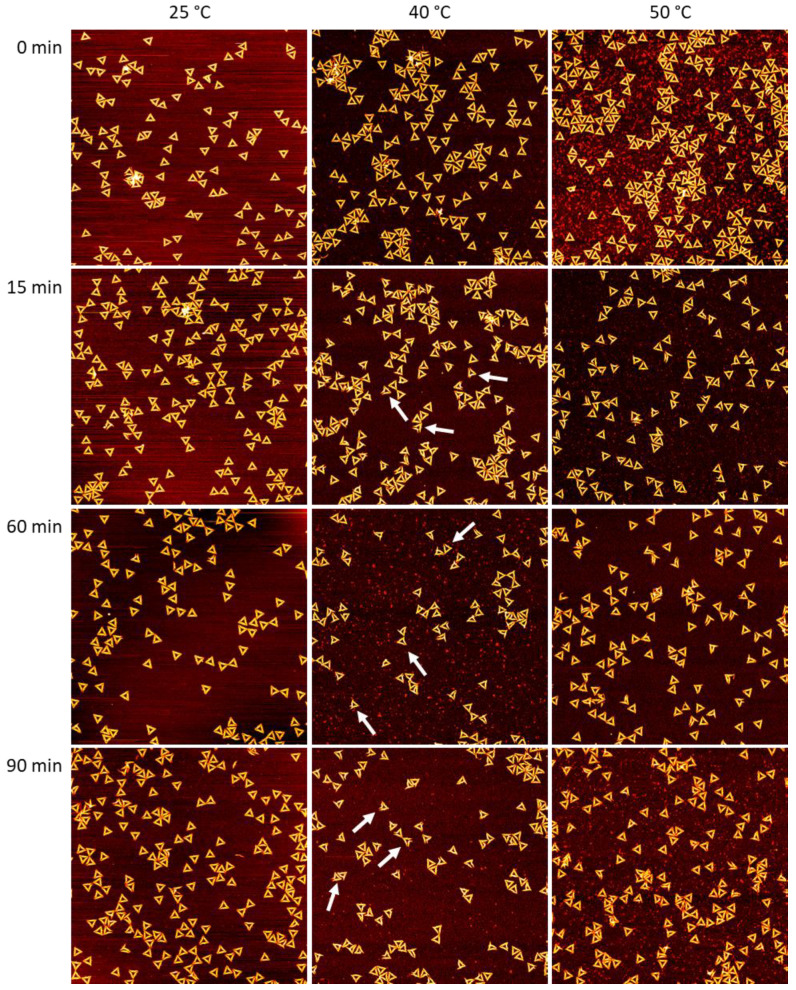
AFM images of DNA origami triangles after incubation in 2 M Gdm_2_SO_4_ at different times and temperatures. Images have a size and height scale of 3 × 3 µm^2^ and 2 nm, respectively. The white arrows indicate damaged yet mostly intact triangles with one or two ruptured vertices. For additional AFM images, see Figures S4–S6.

Gdm_2_SO_4_ is more complex in its effect on DNA origami nanostructures, as it pairs the chaotropic Gdm^+^ cation with the kosmotropic SO_4_^2−^ anion [30]. As can be seen in Figure 3, a rather similar behavior as for GdmCl was observed, with moderate DNA origami damage. This is rather remarkable considering that, at a salt concentration of 2 M, the concentration of the denaturing Gdm^+^ cations was twice as high as for GdmCl. Furthermore, the apparent time dependencies and the type of damage were similar to the case of GdmCl. In particular, almost all damaged DNA origami triangles had ruptured vertices but intact trapezoids.

**Figure 4:**
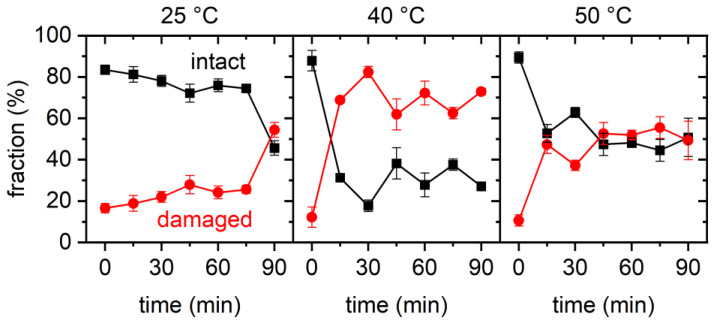
Results of the statistical analysis of the AFM images for 2 M Gdm_2_SO_4_. Each data point represents the average of three AFM images with the standard deviations given as error bars.

Despite all those qualitatively similar observations, the results of the statistical analyses shown in Figure 4 reveal some rather astonishing differences. At 25 °C, we observed a weak yet rather continuous decrease in the fraction of intact DNA origami triangles from about 85% at 0 min to about 75% at 75 min. Then, however, the fraction suddenly dropped to about 45%. Since this was a rather large drop compared to the random fluctuations observed in this and the other datasets, it indeed indicated the onset of a second and more drastic denaturation regime. At the other temperatures of 40 °C and 50 °C, different time dependencies were observed that showed only a large initial drop in the fraction of intact DNA origami triangles between 0 and 15 min, while, at longer incubation times, the fraction saturated and displayed only random fluctuations.

Another interesting feature visible in the plots of Figure 4 is the apparent non-monotonous temperature dependence. At 25 °C, the final value of the fraction of intact DNA origami at 90 min incubation was 46%. Increasing the temperature to 40 °C resulted in a fraction of intact DNA origami triangles of only 29.7% ± 1.9% (averaged over all data points between 15 and 90 min). Such a decrease is to be expected upon an increase in temperature and in agreement with previous observations [30]. However, upon increasing the temperature further to 50 °C, the average value of the fraction of intact DNA origami recovered to 53.3% ± 3.3%. Here, the actual degree of damage was essentially the same as at 40 °C, only with fewer DNA origami triangles with ruptured vertices observed at 50 °C. While such a non-monotonous temperature dependence is rather counterintuitive, a similar behavior was previously observed for GdmCl [30].

From these experiments, we can conclude that rapid DNA origami denaturation within the first 15 min of incubation is a common feature in both GdmCl and Gdm_2_SO_4_, at least under moderately to strongly denaturing conditions. Under weakly denaturing conditions such as 2 M Gdm_2_SO_4_ at 25 °C, however, denaturation occurred more slowly over a time course of more than 1 h. Furthermore, a non-monotonous dependence of DNA origami denaturation in Gdm_2_SO_4_ was observed, which extends previous observations made within a smaller range of temperatures.

### 2.3. Guanidinium Thiocyanate (GdmSCN)

**Figure 5:**
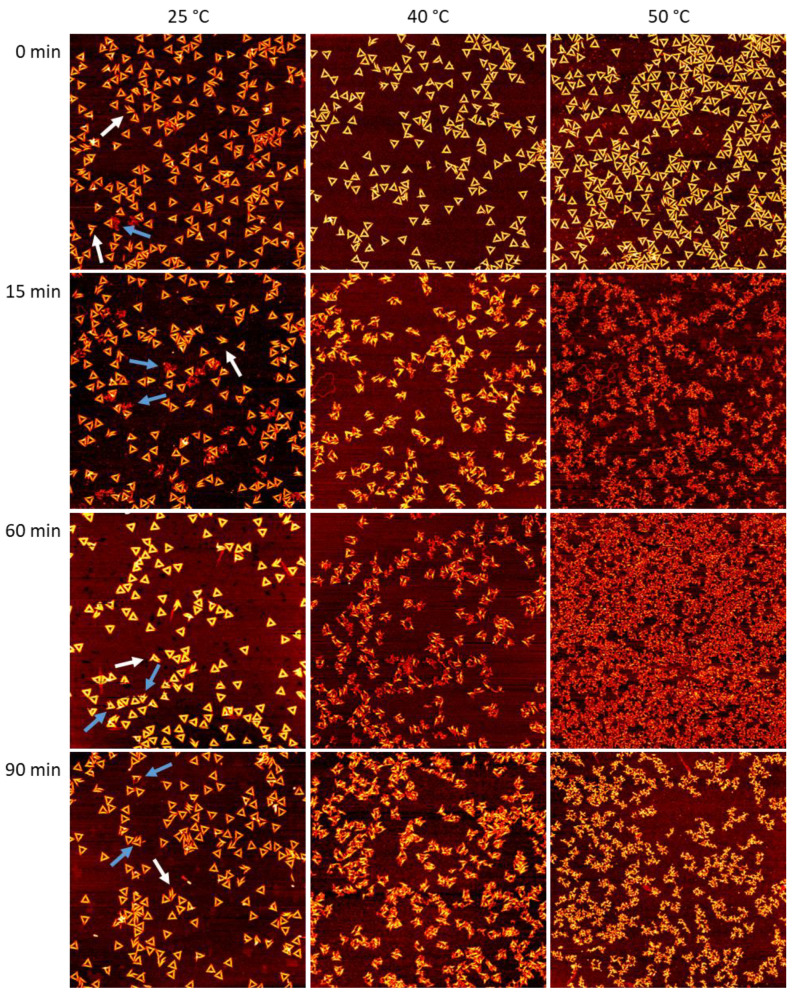
AFM images of DNA origami triangles after incubation in 2 M GdmSCN at different times and temperatures. Images have a size and height scale of 3 × 3 µm^2^ and 2 nm, respectively. The white arrows indicate collapsed triangles with ruptured vertices, while blue arrows indicate triangles with damaged trapezoids or dangling scaffold. For additional AFM images, see Figures S7–S9.

As the third Gdm salt to be investigated in this study, we selected GdmSCN, which is well known as a strong protein denaturant that even exceeds GdmCl in its potency [34,39]. This remarkable denaturant activity results from the pairing of two strongly chaotropic ions, i.e., Gdm^+^ and SCN^−^, which are exceptionally weakly hydrated and, thus, interact very strongly with protein surfaces [46]. In accordance with this behavior, the AFM images in Figure 5 reveal strongly enhanced DNA origami denaturation compared to Gdm_2_SO_4_ and GdmCl. Already at 25 °C, many collapsed triangles with ruptured vertices could be found (white arrows). In addition, some DNA origami had damaged trapezoids or even melted scaffold dangling from them (blue arrows). Most notably, this kind of damage was already apparent at 0 min. At 40 °C, more severe damage was observed. For incubation of 15 min and beyond, virtually all DNA origami nanostructures were severely damaged and had a completely collapsed and partially melted appearance. Increasing the temperature further to 50 °C resulted in the complete denaturation of all the DNA origami triangles, such that only unstructured scaffold was found at the mica surface.

**Figure 6:**
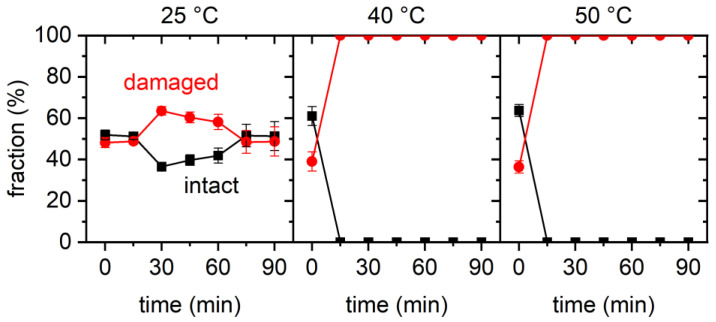
Results of the statistical analysis of the AFM images for 2 M GdmSCN. Each data point represents the average of three AFM images with the standard deviations given as error bars.

Interestingly, the results of the statistical analysis of the AFM images shown in Figure 6 reveal that the fraction of intact DNA origami at 25 °C remained roughly constant throughout the time course of the experiment and fluctuated around a value of about 46%. This is rather remarkable since, for all experiments with GdmCl and Gdm_2_SO_4_ described above, fractions of intact DNA origami of about 80% to 90% were obtained at 0 min incubation. In contrast, exposure to GdmSCN resulted in an immediate destabilization of DNA origami structure at room temperature. Further incubation at 25 °C did not appear to lead to additional denaturation. At both 40 °C and 50 °C, however, incubation for an additional 15 min resulted in the fraction of intact DNA origami dropping to 0%.

## 3. Discussion

**Figure 7:**
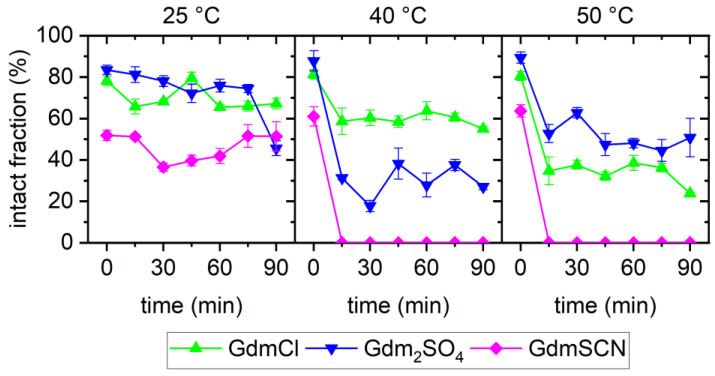
Comparison of the fractions of intact DNA origami triangles obtained in the different Gdm salts at different temperatures and as a function of incubation time.

Figure 7 directly compares the fractions of intact DNA origami nanostructures during exposure to the different Gdm salts as a function of incubation time. As can be seen, for all temperatures investigated in the present work, GdmSCN was the strongest DNA origami denaturant, which is in agreement with its known effect on protein structure [34,39,46]. In particular, exposure to GdmSCN resulted in immediate denaturation, such that a strongly reduced fraction of intact DNA origami was already observed at 0 min. Most remarkably, GdmSCN was the only salt that achieved complete melting of the DNA origami triangles at a concentration as low as 2 M. This can be seen in the AFM images in Figure 5, which show only unstructured scaffolds after 15 min of incubation at 50 °C, whereas, after incubation under equivalent conditions in the other salts, many intact triangles could still be observed (see Figure 1 and Figure 3). In GdmCl, complete DNA origami denaturation has so far only been observed at a concentration of 6 M and incubation temperatures of 37 °C or higher [28], which demonstrates the high denaturant activity of GdmSCN already at comparably low concentrations. This makes GdmSCN a promising candidate for applications that require efficient DNA origami denaturation at low temperatures, for instance, in molecular lithography [47,48].

With regard to the time dependence of DNA origami denaturation, it appears that most denaturation occurred within the first 15 min of incubation, after which the induced DNA origami damage saturated in terms of both the fraction of damaged DNA origami and the degree of damage. The only exception to this behavior was observed for Gdm_2_SO_4_ at 25 °C. Under such weakly denaturing conditions, the fraction of intact DNA origami triangles decreased only weakly during the first 75 min, after which a sudden drop from about 75% to about 45% occurred. A similar behavior was previously observed for DNA origami triangles subjected to multiple freeze–thaw cycles and was attributed to the gradual accumulation of DNA damage [12]. In the present case, the data suggest the gradual build-up of unstacked duplexes and dissociated base pairs in the DNA origami, which, upon reaching a certain threshold, results in the sudden collapse of the DNA origami shape, mostly by dissociation at the particularly vulnerable vertices [28,41]. Indeed, close inspection of the corresponding AFM image in Figure 3 revealed that the vast majority of damaged triangles under this condition had one or two dissociated vertices but intact trapezoids. Under more strongly denaturing conditions, this accumulation of damage seems to happen much faster, such that the maximum degree of damage was already observed after 15 min of incubation.

Lastly, different dependencies of DNA origami denaturation on incubation temperature were observed for the different salts. For GdmCl and GdmSCN, the fraction of intact DNA origami decreased with increasing temperature. For 2 M GdmCl, this observation is in fair agreement with previously reported results [28,30]. For Gdm_2_SO_4_, however, a non-monotonous temperature dependence was observed, in which the fraction of intact DNA origami first decreased with temperature in the range from 25 °C to 40 °C, after which it increased again between 40 °C and 50 °C. A non-monotonous temperature dependence of the fraction of intact DNA origami was previously observed for 4 M GdmCl, where an increase was observed between 23 °C and 30 °C, followed by a decrease between 37 °C and 42 °C. This was explained by DNA origami denaturation in Gdm salts being governed by heat capacity changes, with the free enthalpy of reaction becoming a non-monotonous function of temperature, which could be reproduced with a thermodynamic model [30]. The current results for 2 M Gdm_2_SO_4_, however, do not agree with the predictions of this model, which was derived on the basis of previous experimental observations. Even though the initial decrease in the fraction of intact DNA origami between 25 °C and 40 °C is well reproduced by the model, it also predicts that the intact fraction will monotonously decrease with increasing temperature [30]. This discrepancy might be caused by the limited temperature range of the AFM investigations of 23 to 42 °C, which was used to prime the analysis of the CD spectra and derive the thermodynamic model of Gdm^+^ denaturation. In addition, the CD spectra were recorded within a temperature ramp, which is very different from the isothermal conditions used in the present study. However, further investigations are needed to resolve this issue, particularly in a wider temperature range and while considering temperature-specific variations in denaturation kinetics.

In summary, our results underscore the exceptionally high complexity of DNA origami denaturation in Gdm salts and highlight the necessity of more detailed experimental and theoretical studies that explore the complete parameter space of the underlying reactions. Furthermore, future studies should also assess possible influences of DNA origami shape, superstructure, and local and global design features. While several previous studies found that such factors may dramatically alter the interactions between the DNA origami nanostructures and various molecules and cations [1,2,3,17,19,20,21,24,25], similar superstructure-dependent effects in DNA origami denaturation by Gdm salts are largely unexplored. Nevertheless, on the basis of the mentioned studies, we also anticipate a strong influence of DNA origami shape and superstructure on the denaturing effect of Gdm salts. This, in particular, concerns bulky 3D DNA origami nanostructures, which were previously found to be more sensitive toward ion-binding effects because of their dense packing of helices and the associated higher importance of electrostatic repulsion between neighboring core helices [1]. We can only speculate, however, whether these differences in the electrostatic interactions can also influence any anion-specific effects. Lastly, while previous studies demonstrated that GdmCl denaturation of DNA origami nanostructures shows rather different effects and dependencies than urea denaturation [28,29], it remains to be seen whether these effects and dependencies are also observed for other chaotropic salts such as other thiocyanate or tetrapopylammonium salts. Corresponding investigations aimed at elucidating these issues are currently underway.

## 4. Materials and Methods

### 4.1. DNA Origami Synthesis and Purification

DNA origami triangles [40] were assembled as previously described [30] using the 7249 nt M13mp18 scaffold (Tilibit GmbH, München, Germany) and about 200 staple strands (Eurofins Genomics GmbH, Ebersberg, Germany) in 10 mM Tris buffer (Sigma-Aldrich Chemie GmbH, Taufkirchen, Germany) containing 10 mM MgAc_2_ (Sigma-Aldrich Chemie GmbH, Taufkirchen, Germany). The pH of the Tris buffer was adjusted to 8.0 with acetic acid (Merck KGaA, Darmstadt, Germany). After thermal annealing in a Primus 25 advanced thermocycler (PEQLAB Biotechnologie GmbH, Erlangen, Germany), the DNA origami triangles were purified by PEG precipitation [30]. The precipitate was redissolved in Tris/MgAc_2_ buffer overnight, after which the DNA origami concentration was determined using an Implen Nanophotometer P330 (Implen GmbH, München, Germany) and adjusted to 100 nM with Tris/MgAc_2_ buffer.

### 4.2. Guanidinium Exposure

First, 100 µL samples were prepared by mixing 9.5 μL of 100 mM Tris buffer containing 100 mM MgAc_2_, 5 μL of 100 nM DNA origami solution, and 85.5 µL of 2.34 M Gdm salt solution to yield a DNA origami and Gdm salt concentration of 5 nM and 2 M, respectively. GdmCl and Gdm_2_SO_4_ solutions were prepared by dissolving dry GdmCl (≥99.5%, VWR International S.A.S., Fontenay-sous-Bois, France) and Gdm_2_SO_4_ (99%, Sigma-Aldrich Chemie GmbH, Taufkirchen, Germany) in HPLC-grade water (VWR International S.A.S., Fontenay-sous-Bois, France). For GdmSCN, a 6 M solution (≥99%, Sigma-Aldrich Chemie GmbH, Taufkirchen, Germany) was diluted with HPLC-grade water to the desired concentration of 2.34 M. The samples were vortexed and incubated for 90 min at the desired temperature using a thermocycler PEQLAB Primus 25 advanced. At 15 min intervals, 1 µL aliquots were removed from the samples and deposited immediately on freshly cleaved mica. After addition of 100 µL of 10 mM Tris/MgAc_2_ buffer, which is supposed to prevent any further denaturation, the DNA origami nanostructures were left to adsorb for 5 min, after which the mica surfaces were rinsed with 12 mL of HPLC-grade water and dried in a stream of ultrapure air. For each sample, an additional aliquot was analyzed at 0 min incubation, i.e., directly after mixing the DNA origami with the Gdm salts at room temperature and before heating them to the desired temperature. Each of the time dependencies shown in Figure 2, Figure 4 and Figure 6 was compiled from up to four independent samples.

### 4.3. AFM Imaging

The dry samples were imaged in air using a Bruker Dimension ICON (Bruker France S.A.S., Wissembourg, France) in ScanAsyst Peak-Force Tapping mode with ScanAsyst-Air cantilevers (Bruker AFM Probes, Camarillo, CA, USA) and a JPK Nanowizard III (JPK Instruments, Berlin, Germany) in intermittent contact mode with HQ:NSC18/Al BS cantilevers (MikroMasch, Wetzlar, Germany). The obtained AFM images were flattened and height-adjusted using Gwyddion 2.52 open-source software [49].

### 4.4. Quantification and Statistical Analysis

The fractions of intact and damaged DNA origami triangles visible in the AFM images were determined by visual inspection and manual counting as previously described [1,11]. For each data point in Figure 2, Figure 4 and Figure 6, three images (3 × 3 µm^2^) recorded at different positions on the mica surface were analyzed, with the total number of DNA origami per data point ranging from 225 to 1297. Mean values and standard deviations were computed using OriginPro 2020 (OriginLab Corporation, Northampton, MA, USA).

## Data Availability

The data presented in this study are available on request from the corresponding author.

## Supplementary Materials

The following supporting information can be downloaded at: https://www.mdpi.com/article/10.3390/ijms23158547/s1.

## References

[1] Kielar C., Xin Y., Shen B., Kostiainen M.A., Grundmeier G., Linko V., Keller A. (2018). On the Stability of DNA Origami Nanostructures in Low-Magnesium Buffers. Angew. Chem. Int. Ed. Engl..

[2] Ramakrishnan S., Shen B., Kostiainen M.A., Grundmeier G., Keller A., Linko V. (2019). Real-Time Observation of Superstructure-Dependent DNA Origami Digestion by DNase I Using High-Speed Atomic Force Microscopy. ChemBioChem.

[3] Xin Y., Piskunen P., Suma A., Li C., Ijäs H., Ojasalo S., Seitz I., Kostiainen M.A., Grundmeier G., Linko V. (2022). Environment-Dependent Stability and Mechanical Properties of DNA Origami Six-Helix Bundles with Different Crossover Spacings. Small.

[4] Hahn J., Wickham S.F.J., Shih W.M., Perrault S.D. (2014). Addressing the instability of DNA nanostructures in tissue culture. ACS Nano.

[5] Mei Q., Wei X., Su F., Liu Y., Youngbull C., Johnson R., Lindsay S., Yan H., Meldrum D. (2011). Stability of DNA origami nanoarrays in cell lysate. Nano Lett..

[6] Castro C.E., Kilchherr F., Kim D.-N., Shiao E.L., Wauer T., Wortmann P., Bathe M., Dietz H. (2011). A primer to scaffolded DNA origami. Nat. Methods.

[7] Jiang Z., Zhang S., Yang C., Kjems J., Huang Y., Besenbacher F., Dong M. (2015). Serum-induced degradation of 3D DNA box origami observed with high-speed atomic force microscopy. Nano Res..

[8] Pillers M.A., Lieberman M. (2014). Thermal stability of DNA origami on mica. J. Vac. Sci. Technol. B.

[9] Linko V., Shen B., Tapio K., Toppari J.J., Kostiainen M.A., Tuukkanen S. (2015). One-step large-scale deposition of salt-free DNA origami nanostructures. Sci. Rep..

[10] Kim H., Surwade S.P., Powell A., O’Donnell C., Liu H. (2014). Stability of DNA Origami Nanostructure under Diverse Chemical Environments. Chem. Mater..

[11] Kielar C., Xin Y., Xu X., Zhu S., Gorin N., Grundmeier G., Möser C., Smith D.M., Keller A. (2019). Effect of Staple Age on DNA Origami Nanostructure Assembly and Stability. Molecules.

[12] Xin Y., Kielar C., Zhu S., Sikeler C., Xu X., Möser C., Grundmeier G., Liedl T., Heuer-Jungemann A., Smith D.M. (2020). Cryopreservation of DNA Origami Nanostructures. Small.

[13] Zhu B., Zhao Y., Dai J., Wang J., Xing S., Guo L., Chen N., Qu X., Li L., Shen J. (2017). Preservation of DNA Nanostructure Carriers: Effects of Freeze-Thawing and Ionic Strength during Lyophilization and Storage. ACS Appl. Mater. Interfaces.

[14] Fang W., Xie M., Hou X., Liu X., Zuo X., Chao J., Wang L., Fan C., Liu H., Wang L. (2020). DNA Origami Radiometers for Measuring Ultraviolet Exposure. J. Am. Chem. Soc..

[15] Chen H., Li R., Li S., Andréasson J., Choi J.H. (2017). Conformational Effects of UV Light on DNA Origami. J. Am. Chem. Soc..

[16] Sala L., Zerolová A., Rodriguez A., Reimitz D., Davídková M., Ebel K., Bald I., Kočišek J. (2021). Folding DNA into origami nanostructures enhances resistance to ionizing radiation. Nanoscale.

[17] Hanke M., Hansen N., Chen R., Grundmeier G., Fahmy K., Keller A. (2022). Salting-Out of DNA Origami Nanostructures by Ammonium Sulfate. Int. J. Mol. Sci..

[18] Roodhuizen J.A.L., Hendrikx P.J.T.M., Hilbers P.A.J., de Greef T.F.A., Markvoort A.J. (2019). Counterion-Dependent Mechanisms of DNA Origami Nanostructure Stabilization Revealed by Atomistic Molecular Simulation. ACS Nano.

[19] Opherden L., Oertel J., Barkleit A., Fahmy K., Keller A. (2014). Paramagnetic decoration of DNA origami nanostructures by Eu³⁺ coordination. Langmuir.

[20] Ijäs H., Shen B., Heuer-Jungemann A., Keller A., Kostiainen M.A., Liedl T., Ihalainen J.A., Linko V. (2021). Unraveling the interaction between doxorubicin and DNA origami nanostructures for customizable chemotherapeutic drug release. Nucleic Acids Res..

[21] Kollmann F., Ramakrishnan S., Shen B., Grundmeier G., Kostiainen M.A., Linko V., Keller A. (2018). Superstructure-Dependent Loading of DNA Origami Nanostructures with a Groove-Binding Drug. ACS Omega.

[22] Zhao Y.-X., Shaw A., Zeng X., Benson E., Nyström A.M., Högberg B. (2012). DNA origami delivery system for cancer therapy with tunable release properties. ACS Nano.

[23] Hanke M., Gonzalez Orive A., Grundmeier G., Keller A. (2020). Effect of DNA Origami Nanostructures on hIAPP Aggregation. Nanomaterials.

[24] Suma A., Stopar A., Nicholson A.W., Castronovo M., Carnevale V. (2020). Global and local mechanical properties control endonuclease reactivity of a DNA origami nanostructure. Nucleic Acids Res..

[25] Stopar A., Coral L., Di Giacomo S., Adedeji A.F., Castronovo M. (2018). Binary control of enzymatic cleavage of DNA origami by structural antideterminants. Nucleic Acids Res..

[26] Ray A., Liosi K., Ramakrishna S.N., Spencer N.D., Kuzuya A., Yamakoshi Y. (2020). Single-Molecule AFM Study of DNA Damage by 1O2 Generated from Photoexcited C60. J. Phys. Chem. Lett..

[27] Chen H., Zhang H., Pan J., Cha T.-G., Li S., Andréasson J., Choi J.H. (2016). Dynamic and Progressive Control of DNA Origami Conformation by Modulating DNA Helicity with Chemical Adducts. ACS Nano.

[28] Ramakrishnan S., Krainer G., Grundmeier G., Schlierf M., Keller A. (2016). Structural stability of DNA origami nanostructures in the presence of chaotropic agents. Nanoscale.

[29] Ramakrishnan S., Krainer G., Grundmeier G., Schlierf M., Keller A. (2017). Cation-Induced Stabilization and Denaturation of DNA Origami Nanostructures in Urea and Guanidinium Chloride. Small.

[30] Hanke M., Dornbusch D., Hadlich C., Rossberg A., Hansen N., Grundmeier G., Tsushima S., Keller A., Fahmy K. (2022). Anion-specific structure and stability of guanidinium-bound DNA origami. Comput. Struct. Biotechnol. J..

[31] Schellman J.A. (2002). Fifty years of solvent denaturation. Biophys. Chem..

[32] Graziano G. (2011). Contrasting the denaturing effect of guanidinium chloride with the stabilizing effect of guanidinium sulfate. Phys. Chem. Chem. Phys..

[33] Mason P.E., Dempsey C.E., Vrbka L., Heyda J., Brady J.W., Jungwirth P. (2009). Specificity of ion-protein interactions: Complementary and competitive effects of tetrapropylammonium, guanidinium, sulfate, and chloride ions. J. Phys. Chem. B.

[34] Heyda J., Okur H.I., Hladílková J., Rembert K.B., Hunn W., Yang T., Dzubiella J., Jungwirth P., Cremer P.S. (2017). Guanidinium can both Cause and Prevent the Hydrophobic Collapse of Biomacromolecules. J. Am. Chem. Soc..

[35] Ding B., Mukherjee D., Chen J., Gai F. (2017). Do guanidinium and tetrapropylammonium ions specifically interact with aromatic amino acid side chains?. Proc. Natl. Acad. Sci. USA.

[36] Sarkar S., Singh P.C. (2020). Alteration of the groove width of DNA induced by the multimodal hydrogen bonding of denaturants with DNA bases in its grooves affects their stability. Biochim. Biophys. Acta Gen. Subj..

[37] Sarkar S., Singh P.C. (2021). Sequence specific hydrogen bond of DNA with denaturants affects its stability: Spectroscopic and simulation studies. Biochim. Biophys. Acta Gen. Subj..

[38] Zhang Y., Cremer P.S. (2006). Interactions between macromolecules and ions: The Hofmeister series. Curr. Opin. Chem. Biol..

[39] von Hippel P.H., Wong K.-Y. (1965). On the Conformational Stability of Globular Proteins. J. Biol. Chem..

[40] Rothemund P.W.K. (2006). Folding DNA to create nanoscale shapes and patterns. Nature.

[41] Ramakrishnan S., Schärfen L., Hunold K., Fricke S., Grundmeier G., Schlierf M., Keller A., Krainer G. (2019). Enhancing the stability of DNA origami nanostructures: Staple strand redesign versus enzymatic ligation. Nanoscale.

[42] Matković A., Vasić B., Pešić J., Prinz J., Bald I., Milosavljević A.R., Gajić R. (2016). Enhanced structural stability of DNA origami nanostructures by graphene encapsulation. New J. Phys..

[43] Chen Y., Wang P., Xu Y., Li X., Zhu Y., Zhang Y., Zhu J., Huang G., He D. (2018). Different Stability of DNA Origami Nanostructure between on Interface and in Bulk Solution. ACS Appl. Bio Mater..

[44] Chen Y., Wang P., Liu Y., Liu T., Xu Y., Zhu S., Zhu J., Ye K., Huang G., Dannong H. (2018). Stability and recovery of DNA origami structure with cation concentration. Nanotechnology.

[45] Xin Y., Zargariantabrizi A.A., Grundmeier G., Keller A. (2021). Magnesium-Free Immobilization of DNA Origami Nanostructures at Mica Surfaces for Atomic Force Microscopy. Molecules.

[46] Mason P.E., Neilson G.W., Dempsey C.E., Barnes A.C., Cruickshank J.M. (2003). The hydration structure of guanidinium and thiocyanate ions: Implications for protein stability in aqueous solution. Proc. Natl. Acad. Sci. USA.

[47] Gállego I., Manning B., Prades J.D., Mir M., Samitier J., Eritja R. (2017). DNA-Origami-Driven Lithography for Patterning on Gold Surfaces with Sub-10 nm Resolution. Adv. Mater..

[48] Sajfutdinow M., Uhlig K., Prager A., Schneider C., Abel B., Smith D.M. (2017). Nanoscale patterning of self-assembled monolayer (SAM)-functionalised substrates with single molecule contact printing. Nanoscale.

[49] Nečas D., Klapetek P. (2012). Gwyddion: An open-source software for SPM data analysis. Open Phys..

