# Supplementary material for: Time-Dependent DNA Origami Denaturation by Guanidinium Chloride, Guanidinium Sulfate, and Guanidinium Thiocyanate
Source: Int J Mol Sci. 2022 Aug 1;23(15):8547. doi: 10.3390/ijms23158547 (PMC9368935; doi:10.3390/ijms23158547)
Supplement: Supplementary file 1 [file ijms-23-08547-s001.zip › ijms-1816980-supplementary.pdf]

Supplementary Materials

Article

Time-Dependent DNA Origami Denaturation by Guanidinium Chloride, Guanidinium Sulfate, and Guanidinium Thiocyanate

Marcel Hanke †, Niklas Hansen †,‡, Emilia Tomm, Guido Grundmeier and Adrian Keller *

Technical and Macromolecular Chemistry, Paderborn University, Warburger Str. 100, 33098 Paderborn, Germany; marcelha@mail.uni-paderborn.de (M.H.); niklashansen94@aol.de (N.H.); emiliat@mail.uni-paderborn.de (E.T.); g.grundmeier@tc.uni-paderborn.de (G.G.)

* Correspondence: adrian.keller@uni-paderborn.de; Tel.: +49-5251-60-5722

† These authors contributed equally to this work.

‡ Present address: Department of Biophysical Chemistry, J. Heyrovsky Institute of Physical Chemistry, Czech Academy of Sciences, Dolejškova 3, 182 23 Prague 8, Czech Republic.

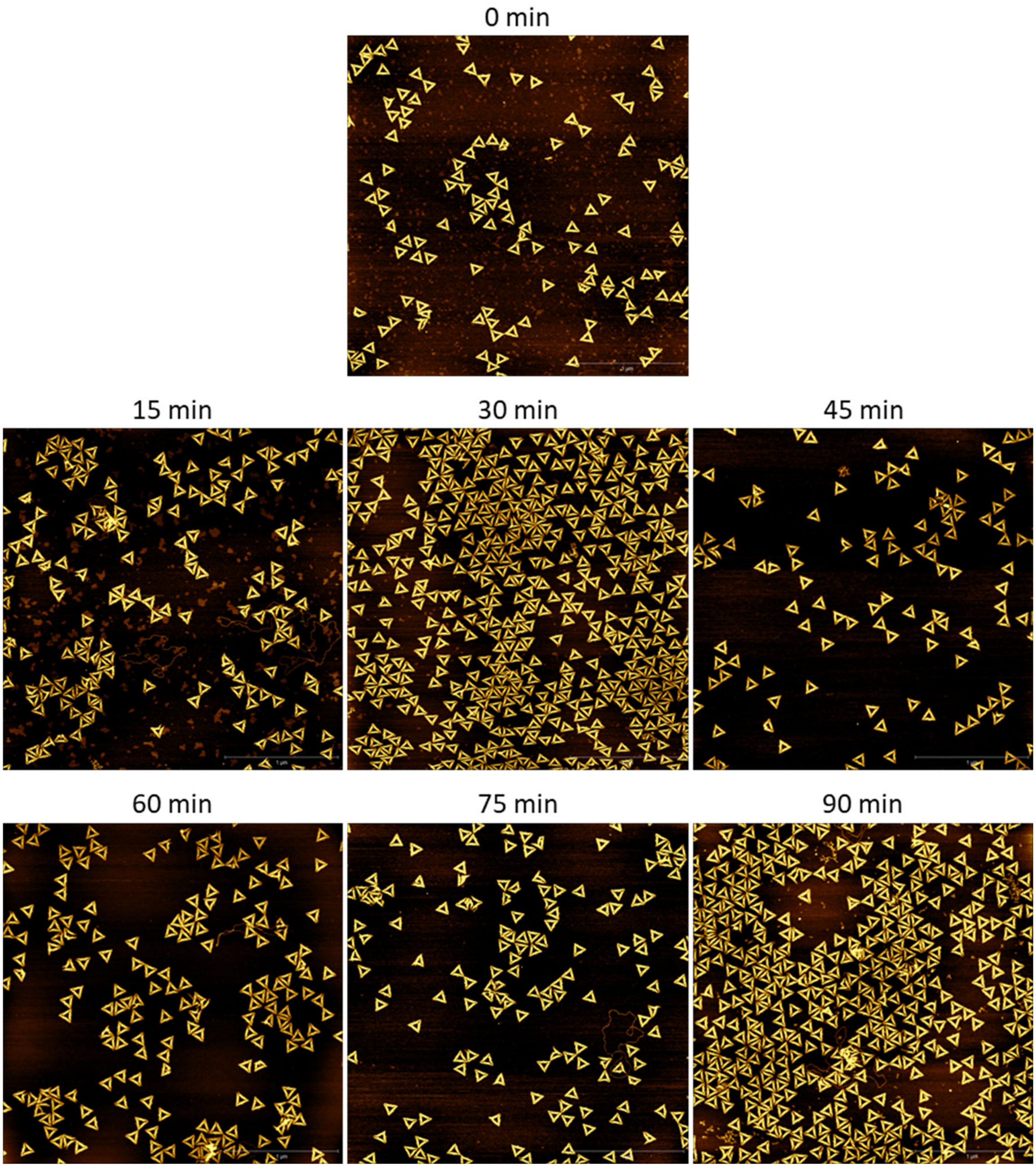

Figure S1: Additional AFM images of DNA origami triangles exposed to GdmCl at 25 °C.

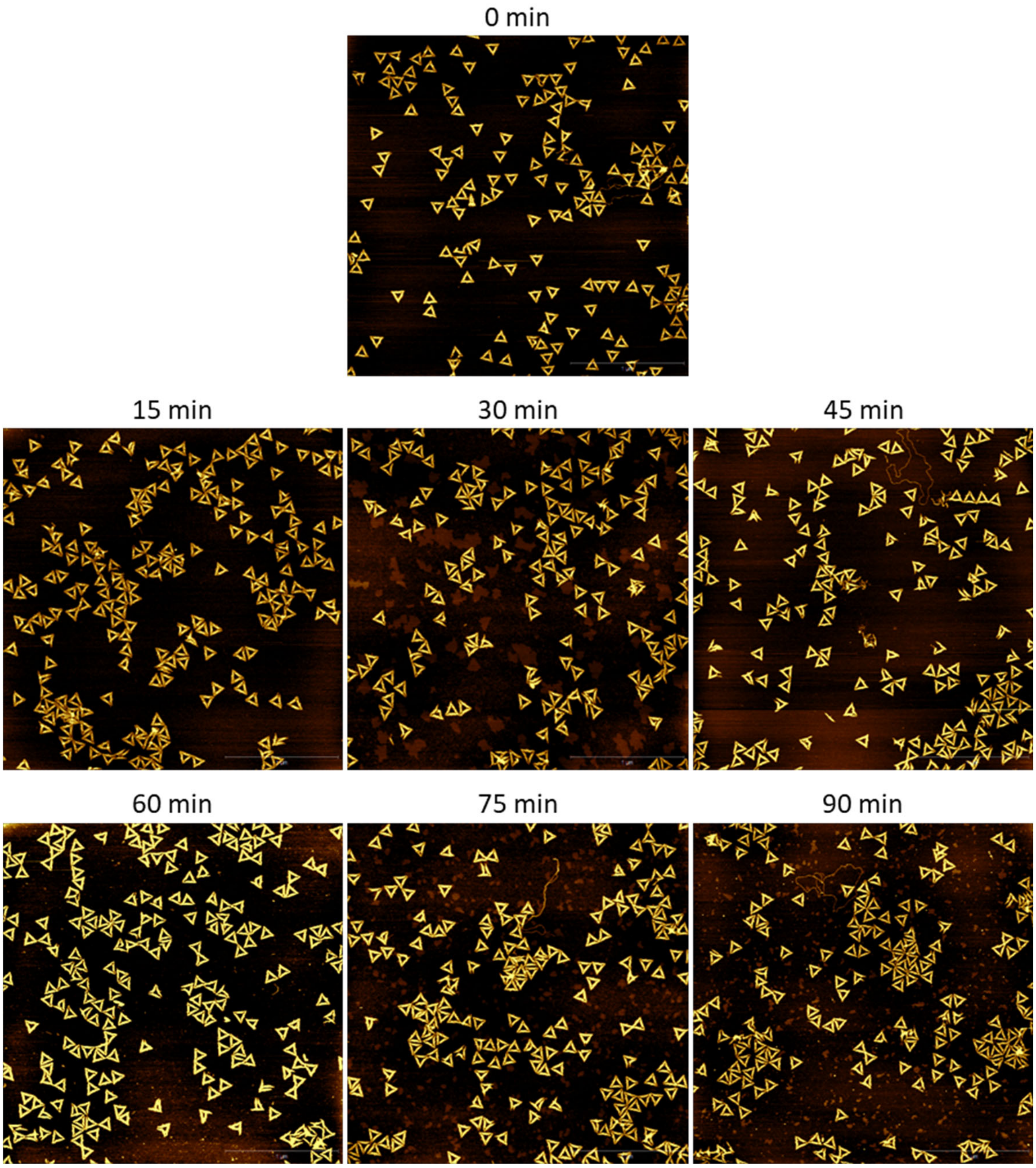

Figure S2: Additional AFM images of DNA origami triangles exposed to GdmCl at 40 °C.

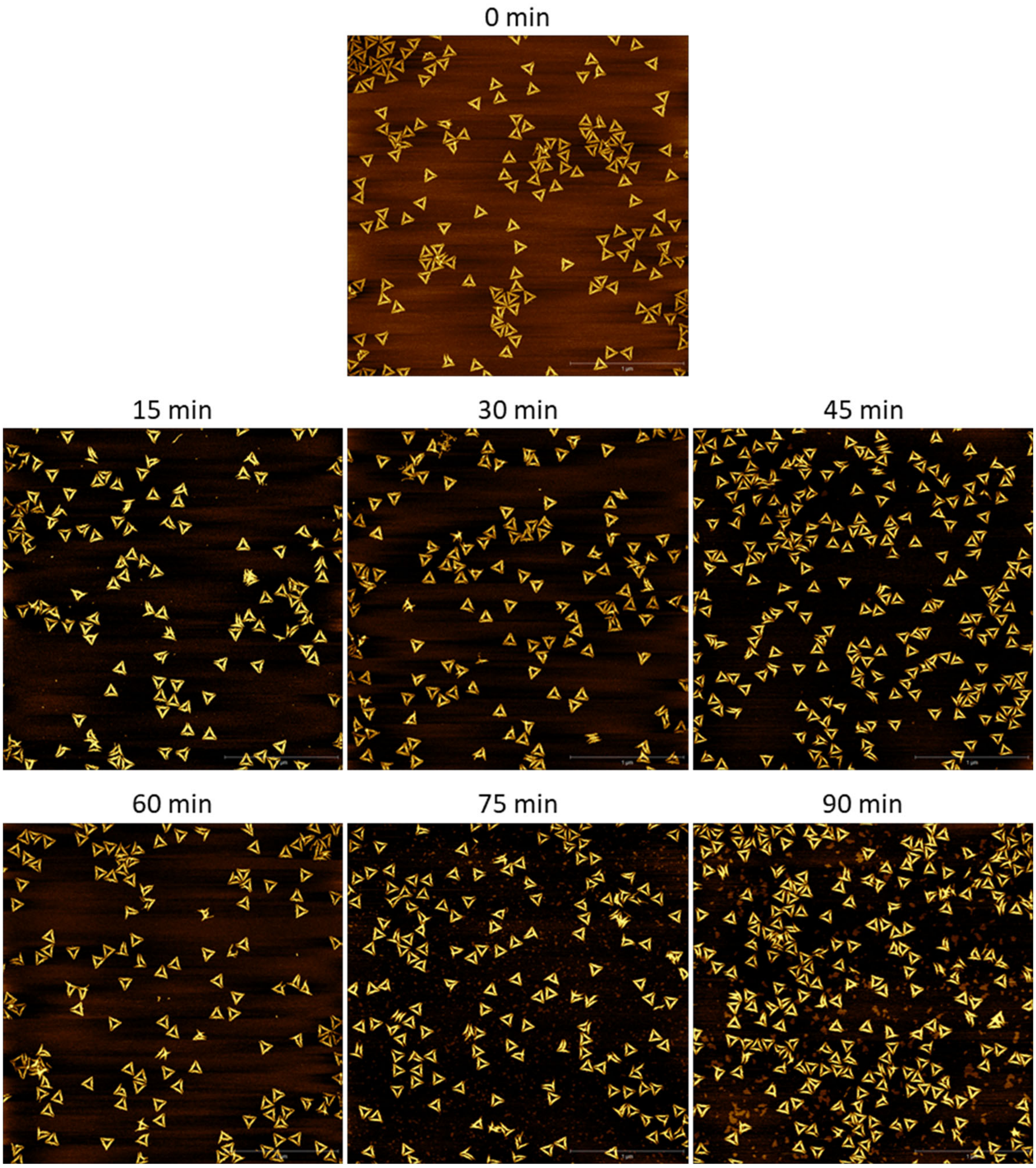

Figure S3: Additional AFM images of DNA origami triangles exposed to GdmCl at 50 °C.

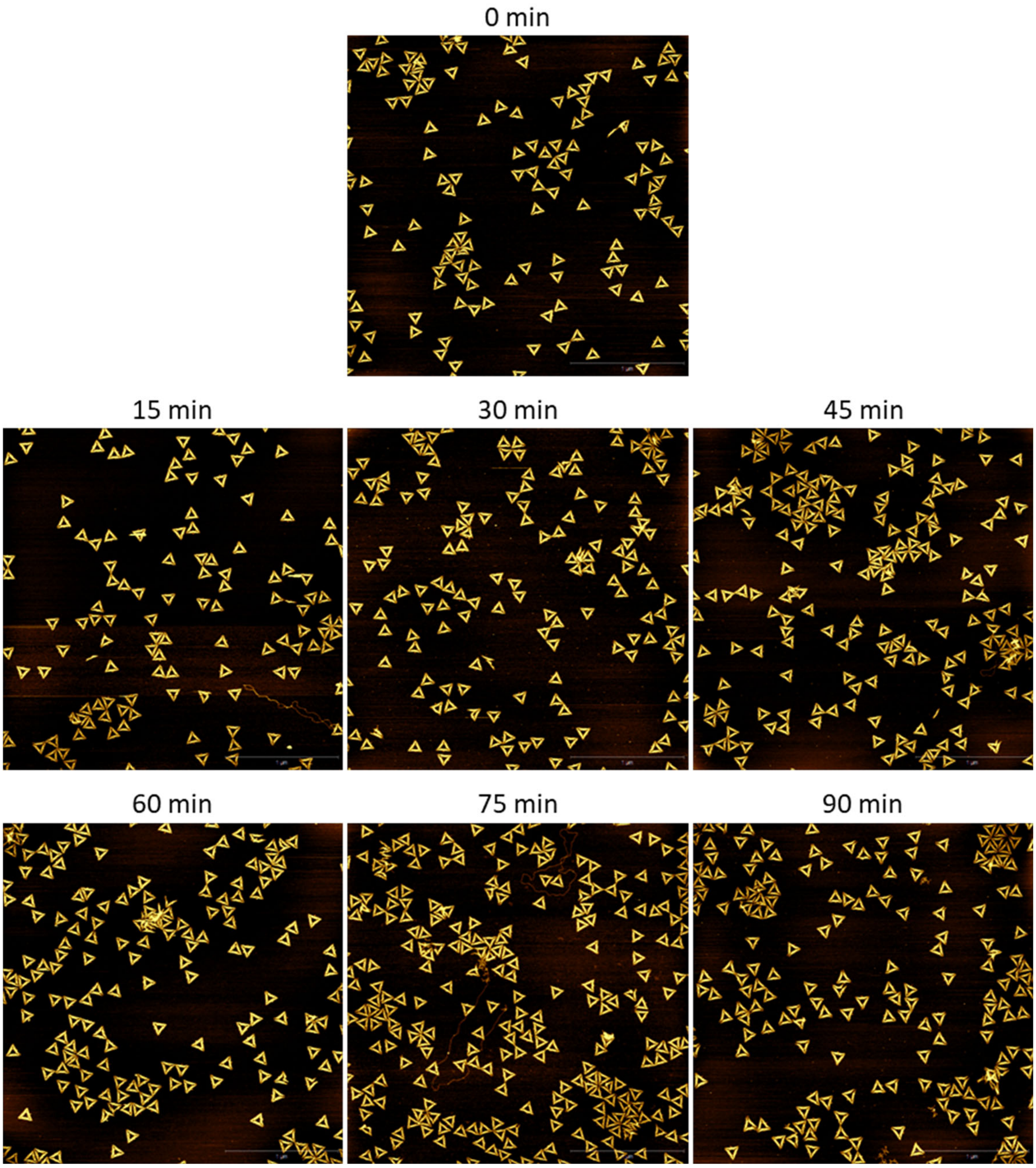

Figure S4: Additional AFM images of DNA origami triangles exposed to Gdm_2SO_4 at 25 °C.

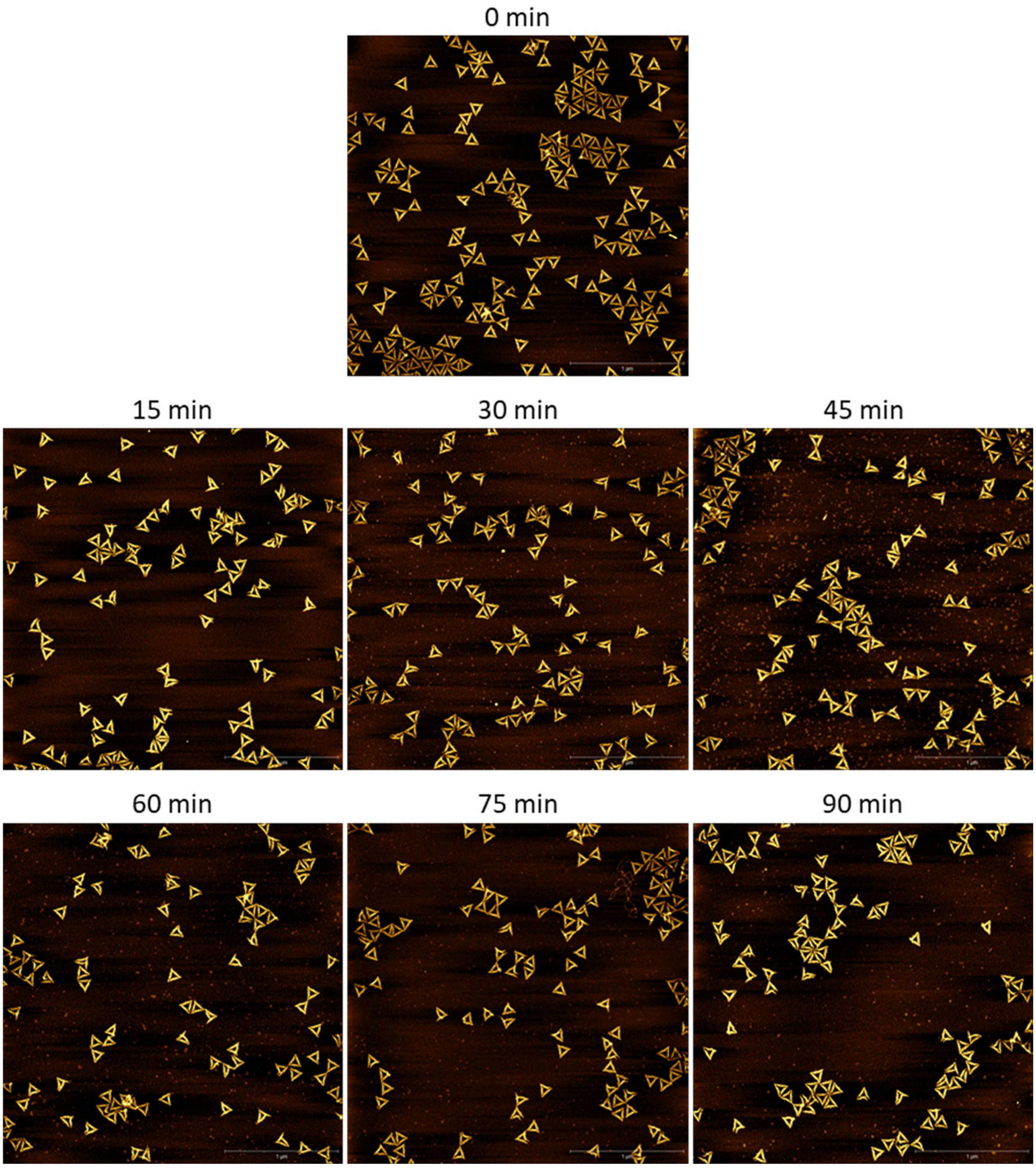

Figure S5: Additional AFM images of DNA origami triangles exposed to Gdm_2SO_4 at 40 °C.

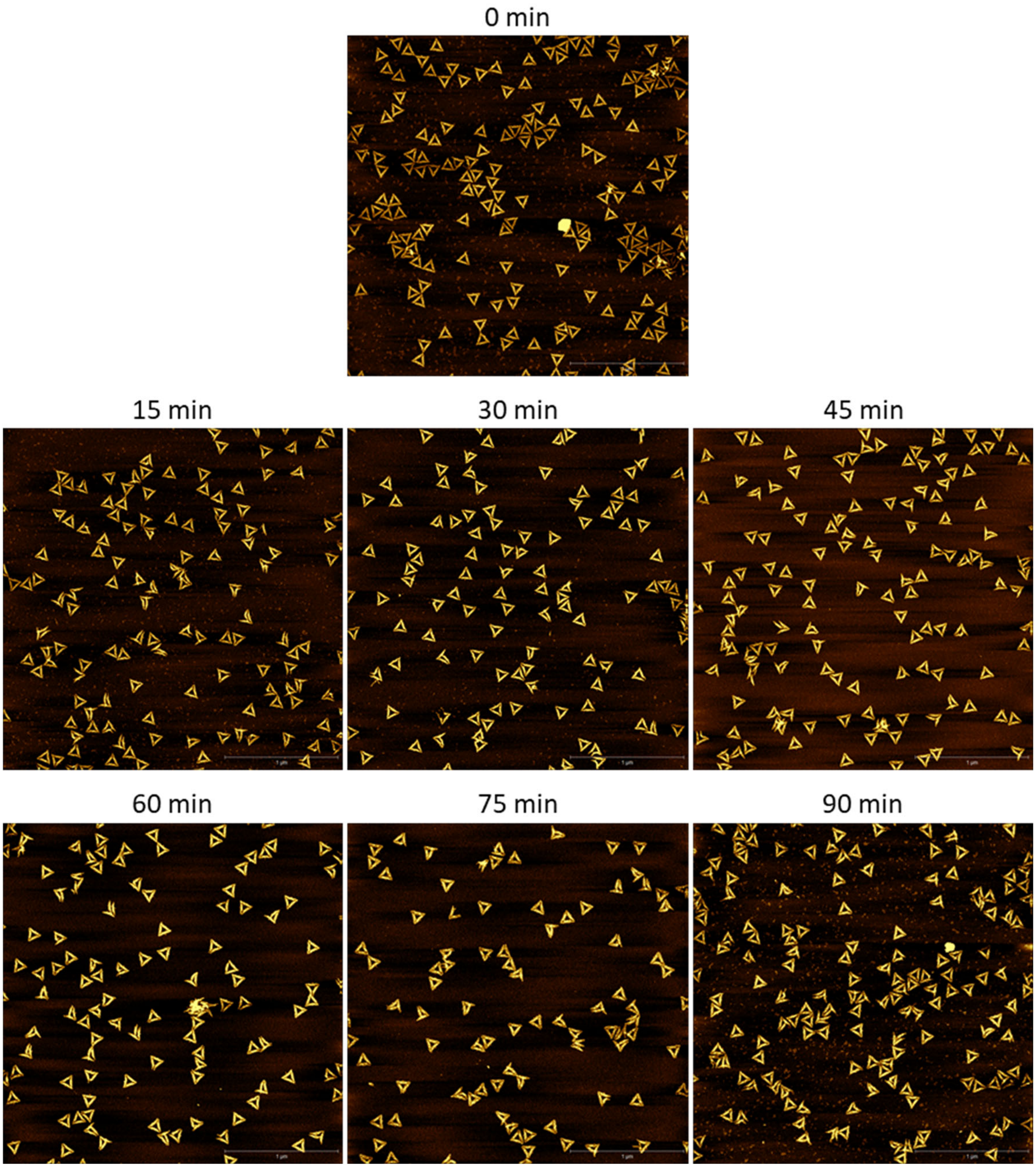

Figure S6: Additional AFM images of DNA origami triangles exposed to Gdm_2SO_4 at 50 °C.

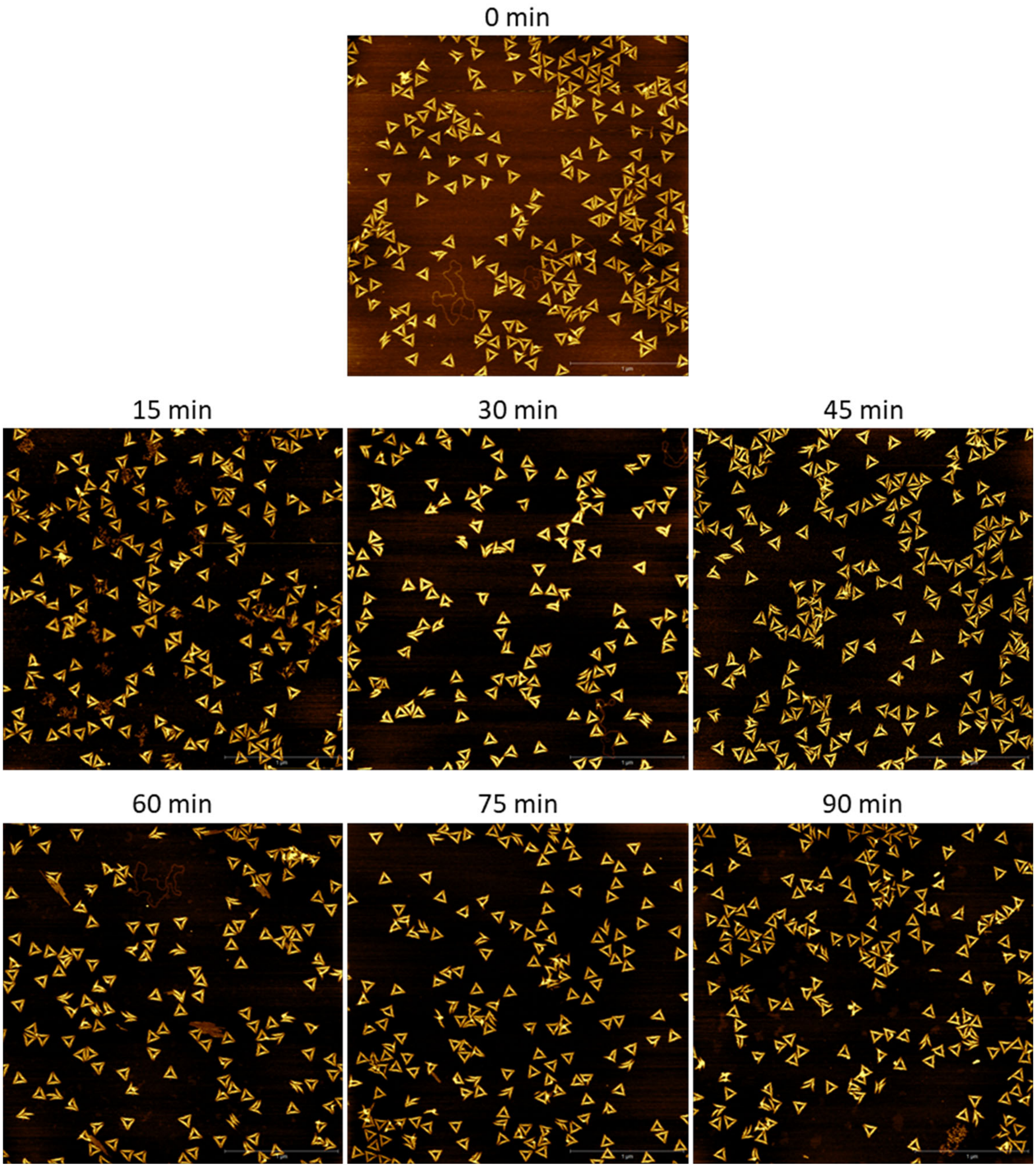

Figure S7: Additional AFM images of DNA origami triangles exposed to GdmSCN at 25 °C.

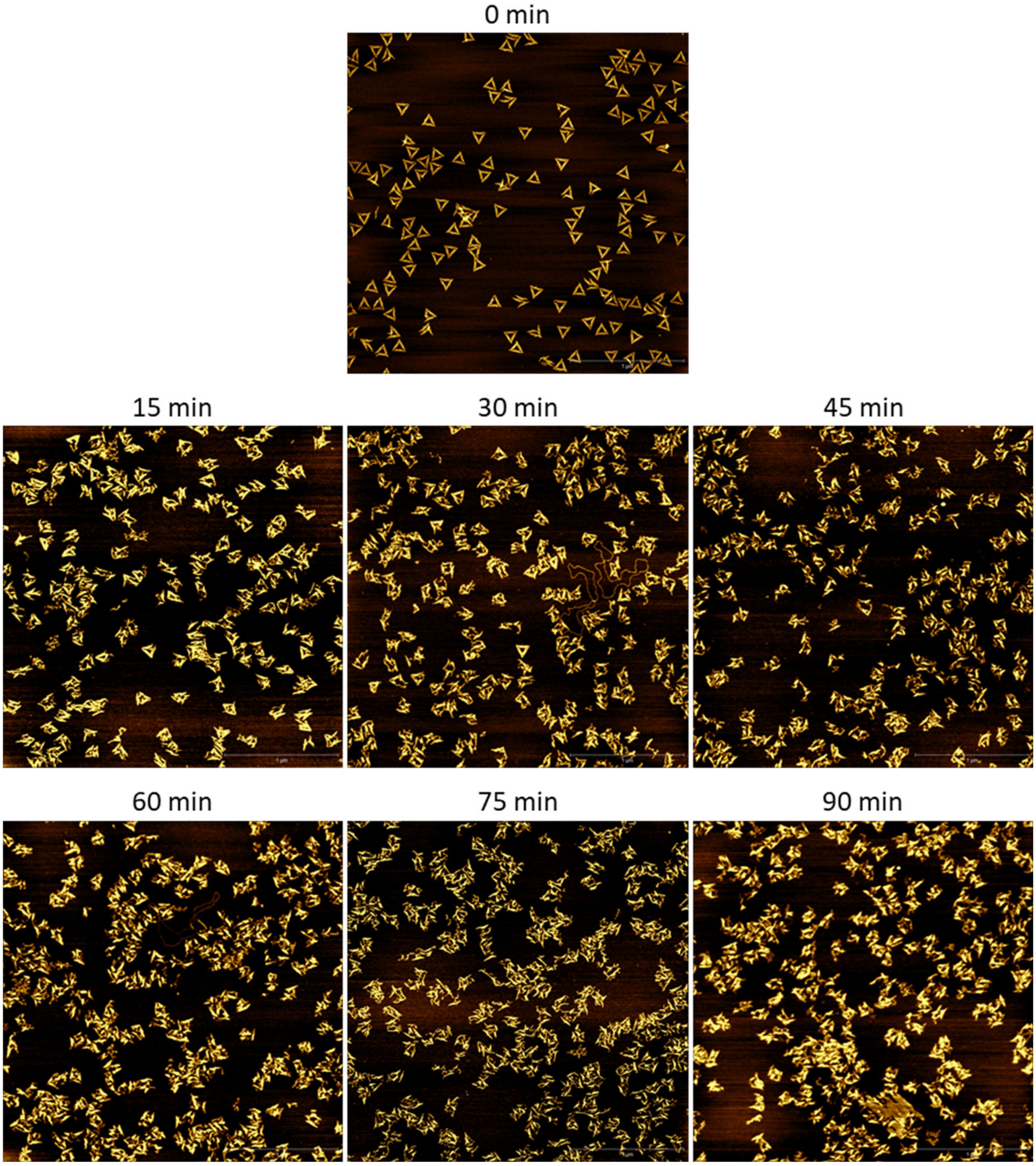

Figure S8: Additional AFM images of DNA origami triangles exposed to GdmSCN at 40 °C.

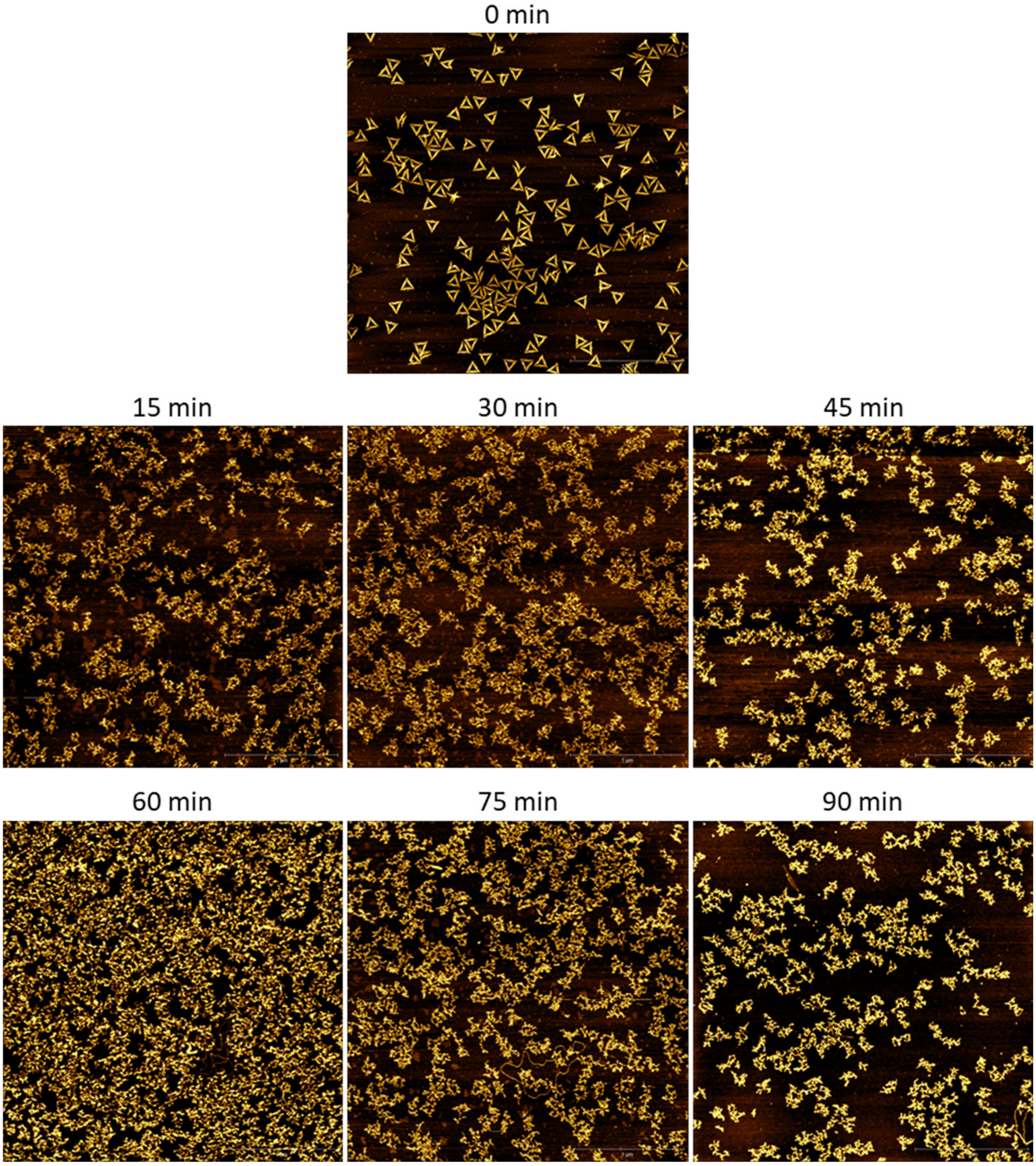

Figure S9: Additional AFM images of DNA origami triangles exposed to GdmSCN at 50 °C.
